# Comparison of Rectal and Gastrointestinal Core Temperatures During Heat Tolerance Testing

**DOI:** 10.3390/medicina61061111

**Published:** 2025-06-19

**Authors:** Melissa J. Crowe, Michael T. Meehan, Rhondda E. Jones

**Affiliations:** 1 College of Medicine and Dentistry, James Cook University, Townsville, QLD 4811, Australia; michael.meehan1@jcu.edu.au; 2 Tropical Australian Academic Health Centre, Townsville, QLD 4811, Australia; rhondda.jones@jcu.edu.au

**Keywords:** exertional heat illness, heat tolerance, body temperature, gastrointestinal tract, military

## Abstract

*Background and Objectives*: Military capability may be reduced in hot environments with individuals at risk of exertional heat stroke (EHS). Heat tolerance testing (HTT) can be used to indicate readiness to return to duty following EHS. HTT traditionally relies on rectal core temperature (T_re_) assessment via a rectal probe. This study investigated the use of gastrointestinal core temperature (T_gi_) as an alternative to T_re_ during HTT. A secondary aim was to compare physiological factors between heat-tolerant and heat-intolerant trials. *Materials and Methods*: Australian Defence Force personnel undergoing HTT following known or suspected heat stroke volunteered (*n* = 23 cases participating in 26 trials) along with 14 controls with no known heat illness history. Confusion matrices enabled comparison of HTT outcome based on T_gi_ and T_re_. The validity of T_gi_ compared to T_re_ during HTT was assessed using correlation and bias. Comparisons between heat-tolerant and intolerant trials were performed using non-parametric tests. *Results*: Although T_gi_ correlated closely with T_re_ (Spearman’s rank correlation ρ = 0.893; median bias 0.2 °C) there was no consistent pattern in the differences between measures. Importantly, the two measures only agreed on heat tolerance outcome in 80% of trials with T_gi_ failing to detect heat intolerance identified by T_re_ in 6 of 8 trials. If T_gi_ was relied upon for diagnostic outcome, return to duty may occur before full recovery. None of the assessed covariates were related to the difference between T_re_ and T_gi_. In addition, resting heart rate and systolic blood pressure were significantly lower and body surface area to mass ratio significantly higher in heat-tolerant compared to intolerant trials. *Conclusions*: It is not recommended to rely on T_gi_ instead of T_re_ during HTT. Resting heart rate and systolic blood pressure findings point to the importance of aerobic exercise in conveying heat tolerance along with body composition.

## 1. Introduction

Extreme environments pose a serious threat to military capability. Performance in extreme heat leads to reduced cardiovascular capacity as blood flow must be diverted to the skin for cooling and sweat losses result in dehydration [1]. These physiological responses reduce the volume of blood available to the working muscle, reducing performance [1]. Continued activity in extreme heat can lead to heat illness and an inability to perform, putting individual soldiers at risk as well as reducing military capability. Climate change is causing an increasing number of hot days and increased frequency and severity of heat waves with 2023 recorded as the hottest year on record [2]. Thus, heat tolerance of military personnel is an increasingly important issue.

In Australia, the majority of Defence Force personnel are stationed in Northern Australia where hot and humid conditions are experienced for much of the year. Therefore, training to maintain fitness and defence capability occurs in these environments which increases the risk of heat illness. Furthermore, deployment to hot/dry or hot/humid environments where it may not be possible to avoid prolonged exposure to environmental conditions or to reduce activity levels, also puts military personnel at risk of heat illness. Heat risk is further exacerbated in military personnel due to high levels of motivation for task completion, both in training and combat [3]. Many military forces have clear guidelines and policies for preventing heat illness including the Australian Defence Force (ADF). However, despite these guidelines, heat illness continues to be an issue in military populations [4].

Heat stroke is the most serious form of heat illness; it can cause multi-organ dysfunction and can be life-threatening if not treated quickly [5]. Return to duty following exertional heat stroke (EHS) is a complex process [6,7] with no single indicator of recovery [8]. However, once pathology has normalized military personnel are usually permitted a graded return to activity. Some military forces around the world, such as the Israeli Defence Force (IDF) and the ADF, utilize a heat tolerance test (HTT) as an indicator of readiness and safety to return to full duties following EHS.

A HTT provides an indication of the body’s ability to thermoregulate, particularly to control core body temperature, whilst exercising in the heat. The most widely used HTT protocol is the Israeli Defence Force (IDF) protocol [9,10,11]. This HTT involves walking at 5 km/h and 2% grade for 120 min under hot/dry conditions (40 °C, 40% relative humidity). The main variables assessed to indicate heat tolerance during the IDF HTT are core temperature and heart rate (HR) with thresholds of 38.5 °C and 150 bpm, respectively, which, if exceeded, indicate heat intolerance [12]. Core temperature is traditionally assessed by rectal probe during the IDF HTT.

Rectal core temperature (T_re_) is widely employed when assessing core body temperature during exercise and is considered a reliable and valid assessment [13,14]. However, disadvantages include a slower response to changes in temperature [15], the need for cabling between the sensor and data logger and lack of social acceptability. Ingestible telemetric gastrointestinal core temperature pills have been shown to provide a valid assessment of core temperature [16,17] and enable core temperature assessment during field activities. Previous studies have indicated close agreement between gastrointestinal temperature (T_gi_) and T_re_ during outdoor team sport exercise [18], intermittent exercise [19], indoor exercise in hot conditions [20], moderate-intensity cycling in thermoneutral and hot conditions [21] and during recovery from a warm weather running road race [22]. However, the use of T_gi_ as an alternative to T_re_ during HTT, when a real-time, valid assessment of core temperature is essential for individuals at risk of heat illness, has not yet been explored.

The IDF HTT protocol has been utilized by the IDF for many years to gauge readiness to return to duty following EHS [9,10,11]. Heat tolerance has been reported to be associated with a number of intrinsic and extrinsic factors [4]. Intrinsic factors pertain to a person’s individual characteristics such as previous heat illness, gender, body composition and aerobic fitness [4]. These factors have implications for recruitment and training in military forces to prevent heat illness.

The aim of this study was to investigate whether T_gi_ could be employed as an alternative to T_re_ assessment during HTT. A secondary aim of the study was to examine differences in physiological factors between heat-tolerant and intolerant HTT trials. It was hypothesised that T_gi_ would provide a valid assessment of core temperature during HTT using T_re_ as the criterion measure and that heat-tolerant trials would show differences in physiological factors compared to heat-intolerant trials.

## 2. Materials and Methods

### 2.1. Participants and Experimental Design

Participants were recruited from ADF personnel who had been referred to the primary investigator (MC) between the years 2015 to 2020 for HTT following a known or suspected incident of heat stroke (cases; *n* = 23; 20 male, 3 female). The ADF policy on Health Management for the Prevention and Treatment of Heat Casualties [23] prior to 2024 when new policy was introduced, required HTT for all ADF members with more severe heat illness and confirmed or suspected heat stroke as part of the return to duty process. Repeat testing is allowed and three of the ADF participants contributed two data sets to this study (2 male, 1 female). ADF members were referred for testing from all over Australia with 70% of the cases from barracks in Darwin and Townsville (both tropical locations with hot and humid conditions for much of the year) and the remaining 30% from subtropical and temperate climate locations, all with warm to hot summers. Time elapsed between the heat stroke event and HTT ranged from 7 to 68 weeks with one outlier referred almost 6 years after the EHS event. This large variation in timing was the result of the varying time taken for Defence members to be referred for testing, which was beyond the control of the researchers. Controls (*n* = 14; 10 male, 4 female) were recruited from the general population who were taking part in a concurrent study on heat tolerance [24]. Controls could not be recruited from the Defence Force because of the risk to careers from a possible heat-intolerant outcome. Controls were therefore recruited from the university (*n* = 10, physically active staff and students) and from the local sports community (*n* = 4, primary sport triathlon). All controls resided in Townsville in tropical North Queensland and had experienced at least one summer in this location. Ethical approval was obtained from the Department of Defence and Veteran Affairs Human Research Ethics Committee (approval numbers LREP-14-021, approval date: 15 January 2015; and 075-18, approval date: 21 January 2019). Clear written and verbal instructions were provided on the test procedure before informed consent was obtained from all participants prior to taking part in the study. All participants underwent a medical prescreening process prior to participation and were screened for any injury or condition that would prevent them from completing the exercise protocol, medications affecting the assessed parameters and for contraindications to ingestion of a gastrointestinal core temperature pill. Exclusion criteria for the gastrointestinal core temperature pill included previous history of known or suspected obstructive disease of the throat, intestines or bowel; inflammatory bowel disease; hypomotility (slow bowel) disorder; previous gastrointestinal surgery (including to the throat, stomach, intestines or bowel); or any problem with the gag reflex either current or in the past. In addition, individuals with body weight of less than 35 kg or who were scheduled to undergo magnetic resonance imaging within 2–3 days of testing were also excluded. General exclusion criteria included pregnant or lactating women; a history of hypertension, malignant hyperthermia, or diabetes; undergoing treatment for a mental disorder; undergoing treatment for anaemia; and using glucose-lowering agents, prednisolone or beta-blockers. All participants were provided instructions on preparing for the test which included avoiding alcohol and strenuous exercise in the 24 h prior to testing and avoiding caffeine on the day of testing. Advice on water consumption to obtain euhydration prior to testing was also provided.

### 2.2. Procedure

#### 2.2.1. Gastrointestinal Core Temperature Pills

The calibration and serial numbers of the gastrointestinal core temperature pills (CorTemp Core Body Temperature Sensors, HQ Inc., Palmetto, FL, USA) were recorded for programming the data monitor (CorTemp Wireless Monitoring System, HQ Inc., Palmetto, FL, USA). The pills were provided to participants the day before testing. Participants were given instructions on the storage and ingestion of the core temperature pills. Pills were not to be stored or kept near metal or magnetic fields including computers or mobile phones. The participants were asked to ingest the pill 6–8 h before testing to allow for transit from the stomach to small intestine to avoid the influence of drinking on sensor temperature.

#### 2.2.2. Baseline Assessments

All participants arrived at the laboratory at the same time of day (8 am) in an effort to reduce the influence of circadian rhythms on starting core body temperature. They wore shorts and a T-shirt with running shoes. Resting HR (Polar T31 Coded Transmitter, Polar Electro Oy, Kempele, Finland) and blood pressure (BP; Aneroid Sphygmomanometer, ALP-K2, Tokyo, Japan) were assessed as part of the medical prescreening process and any individual whose BP exceeded 140/90 mmHg after two consecutive BP measurements were not tested until medical clearance was obtained. Hydration status was assessed via urine specific gravity (USG; Atago Inc., Tokyo, Japan) from a mid-stream urine sample. Any participants with a USG reading above 1.015 were provided additional water to consume and a second USG assessment undertaken after 30–40 min to ensure euhydration. Nude body weight was assessed in a private cubicle using scales accurate to ±50 g (Tanita RD-545, Tanita Corporation, Tokyo, Japan) to facilitate the assessment of sweat rate. Body weight and standing height (assessed using a wall-mounted stadiometer; Seca Model 220, Hamburg, Germany) were used to determine body surface area (BSA) according to Mostella [25]. Resting core temperature was assessed using the gastrointestinal core temperature pill and a rectal probe (RET-1 Rectal Probe, Physitemp Instruments, LLC, Clifton, NJ, USA) inserted by the participant to a depth of 10 cm beyond the anal sphincter.

#### 2.2.3. Heat Tolerance Test

The HTT was conducted according to the IDF HTT protocol [9,10,11,12] inside a climate control chamber set to 40 °C and 40% relative humidity. Time zero assessments were made of HR, T_re_ and T_gi_. Participants walked on a treadmill at 5 km/h and 2% grade for 120 min with an assessment made every 5 min of HR, and rectal and gastrointestinal core pill temperatures. Water was provided ad libitum throughout the test (median (IQR) water consumed was 0.75 (0.52–1.00) L/hr) with the volume consumed used for the calculation of sweat rate (see equation below). The HTT was discontinued if the participants experienced signs of heat illness such as nausea, headaches, weakness, dizziness or if they requested to discontinue the test. Participants were diagnosed as heat-tolerant if their core temperature remained below 38.5 °C and HR below 150 bpm during the HTT [12]. These HTT criteria have been recommended as the most appropriate for the IDF HTT as they have the highest test specificity [26].

#### 2.2.4. Post-Exercise Assessments

Urine-specific gravity was assessed post-exercise. Nude body weight assessment was repeated post-exercise and sweat rate estimated using the pre- and post-exercise body weights and accounting for fluid intake and any urine produced according to the following formula [27]:

$$\mathrm{SR}=\frac{\left(Pre\;exercise\;body\;weight-post\;exercise\;body\;weight\right)+\left(fluid\;ingested\;during\;exercise\right)-(urine\;excreted\;during\;exercise)}{Exercise\;time\;in\;hours}$$

where SR is sweat rate, weight expressed in kg and fluid and urine in litres.

### 2.3. Statistical Analyses

The data were entered into Excel (Microsoft Office Professional Plus 2016) and transferred into R Studio (R Foundation for Statistical Computing, 4.4.1). The T_re_ and T_gi_ temperature measures along with their differences and residuals were each tested for normality using the Shapiro–Wilk test. QI Macros (QI Macros Lean Six Sigma SPC software; KnowWare International Inc., Chicago, IL, USA, Version 2024) were used in Excel to conduct comparisons between the cases and controls and between heat-tolerant and heat-intolerant trials using the Mann–Whitney U test. Paired data including the starting and final T_re_ and T_gi_ temperatures were compared using the Wilcoxon Signed Rank Test with QI Macros. All other data analyses were conducted using R Studio. Fishers’ Exact test was used to examine the difference in the proportion of heat-tolerant participants between the cases and controls. The validity of T_gi_ as a measure of core temperature during HTT was assessed using T_re_ as the criterion measure. These validity measures included correlation and bias (calculated as the difference between the rectal and gastrointestinal core temperature measurements, i.e., T_gi_−T_re_). As the data were not normally distributed, we calculated Spearman’s rank correlation coefficient, reported the median bias and used bootstrapping to estimate bounds of the 95% limits of agreement (LoA). Bootstrapped samples were generated by first sampling participants with replacement (i.e., cluster sampling) and then sub-sampling contiguous temperature measurements for each participant (i.e., block sampling). A confusion matrix was formulated to determine the agreement between the heat tolerance outcomes (i.e., heat-tolerant or heat-intolerant) as determined by the T_re_ and T_gi_ temperatures. Univariate linear regression was conducted to determine the relationship between resting HR, resting SBP, resting DBP, BSA/mass ratio, commencing core temperature, baseline USG and sweat rate and the difference between the commencing T_re_ and T_gi_ temperatures. Data are presented as median (interquartile range; IQR). The significance threshold alpha was set at 0.05.

## 3. Results

The median (IQR) age, height, weight and BSA/mass ratio of the cases and controls are presented in Table 1 with no significant difference between the cases and controls for these characteristics. Using the HTT criteria of T_re_ above 38.5 °C or HR above 150 bpm, 46.2% of the cases and 64.3% of the control participants were heat-intolerant (Table 1). The proportion of heat-intolerant participants was not significantly different between the two groups (Table 1).

### 3.1. Heat Tolerance Test Outcomes Based on Rectal and Gastrointestinal Core Temperatures

Using the 38.5 °C core temperature criterion, the HTT outcome was compared between T_re_ and T_gi_ for all trials using a confusion matrix (Table 2). The confusion matrix indicates that the two core temperature assessment methods agree on heat tolerance status in 32 of 40 trials (80%; *n* = 20 heat-tolerant outcomes and *n*= 12 heat-intolerant outcomes). Where the results of the two assessment methods differed, most (6 of 8) were cases where the gastrointestinal core pill failed to detect intolerance that had been identified by the rectal probe (Table 2).

### 3.2. Comparison of Rectal and Gastrointestinal Temperatures

**Figure 1:**
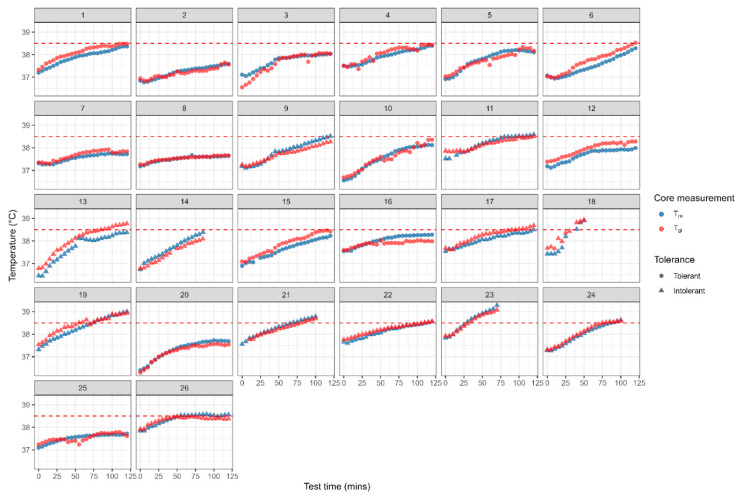
Individual gastrointestinal (T_gi_) and rectal (T_re_) temperature responses during the HTT for cases indicating heat tolerance or intolerance. Dashed line represents the 38.5 °C core temperature criteria for heat tolerance.

**Figure 2:**
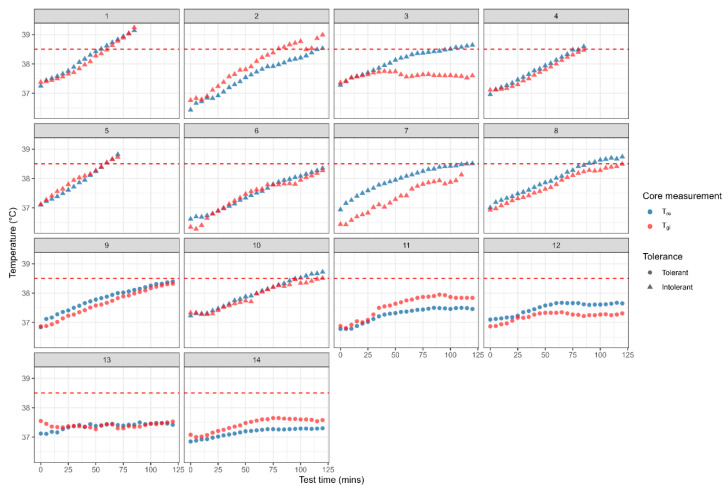
Individual gastrointestinal (T_gi_) and rectal (T_re_) temperature responses during the HTT for controls indicating heat tolerance or intolerance. Dashed line represents the 38.5 °C core temperature criteria for heat tolerance.

The median T_re_ commencing core temperature (37.11, IQR 36.88–37.30 °C) was significantly lower at time zero of the HTT compared to the median T_gi_ (37.24, IQR 36.88–37.44 °C; *p* = 0.003; n = 39 due to one core pill malfunction on the first data point) with 31 of the 39 trials (79.5%) having a lower commencing T_re_. However, the final median core temperature was not significantly different between T_re_ (38.39, IQR 38.02–38.61 °C) and T_gi_ (38.38, IQR 37.92–38.58 °C; *p* = 0.786; n = 39 due to one core pill malfunction on a final data point) with only 18 of the 39 trials (46.2%) showing a lower T_re_ compared to T_gi_ at test completion. Thus, T_re_ was not consistently lower than T_gi_ during the HTT. To illustrate these variable differences individual data sets have been presented. Figure 1 shows the T_re_ and T_gi_ core temperature responses during the HTT for the 26 trials from cases with the 14 controls presented in Figure 2. Note, in 5 HTT for cases, the participants did not finish the full 120 min of the test reaching the core temperature threshold earlier in the test and/or experiencing symptoms of heat illness (Figure 1, trials 14, 18, 21, 23 and 24). Similarly, 3 of the controls did not finish the full 120 min of the HTT for the same reasons (Figure 2, trials 1, 4 and 5).

**Figure 3:**
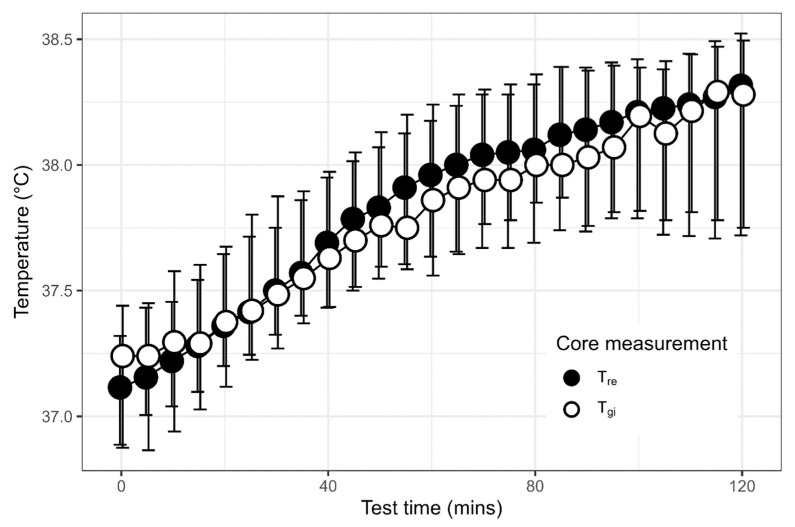
The median (IQR) gastrointestinal (T_gi_) and rectal (T_re_) core temperature measurements for the 120 min of the heat tolerance test (HTT) from all trials. Data points have been offset to aid visualization of the median and IQR values.

**Figure 4:**
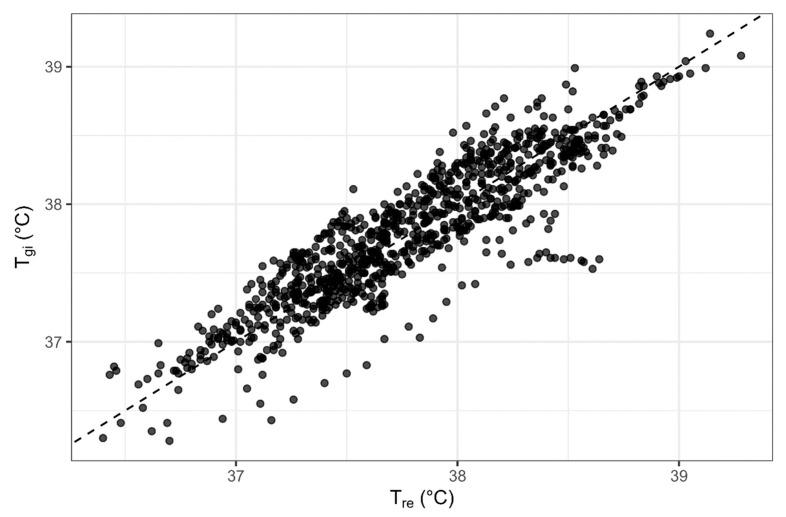
A scatter plot of gastrointestinal (T_gi_) measures against the rectal (T_re_) core temperature measures during the heat tolerance test (HTT) from all trials with a line of equality super-imposed.

**Figure 5:**
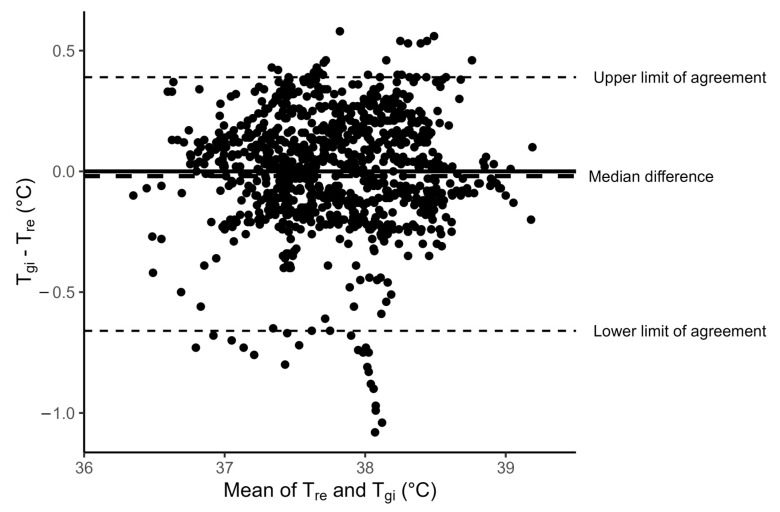
Bland–Altman plot of the difference in the gastrointestinal (T_gi_) and rectal (T_re_) core temperature measurements against the average of the measurements with lines indicating the median difference (bias) and 95% limits of agreement.

The agreement between the two core temperature measurements can be seen in Figure 3 with the median T_re_ and T_gi_ measurements for the 120 min of all HTT trials and Figure 4 with a scatter plot of the T_gi_ measures against the T_re_ measures for all trials. Spearman’s rank correlation coefficient was ρ = 0.893. The median temperature difference (bias) between the two measures was 0.2 °C (95% LoA −0.66 to 0.39 °C) as displayed in the Bland–Altman plot in Figure 5.

### 3.3. Influence of Covariates on Difference Between Rectal and Gastrointesinal Temperatures

**Figure 6:**
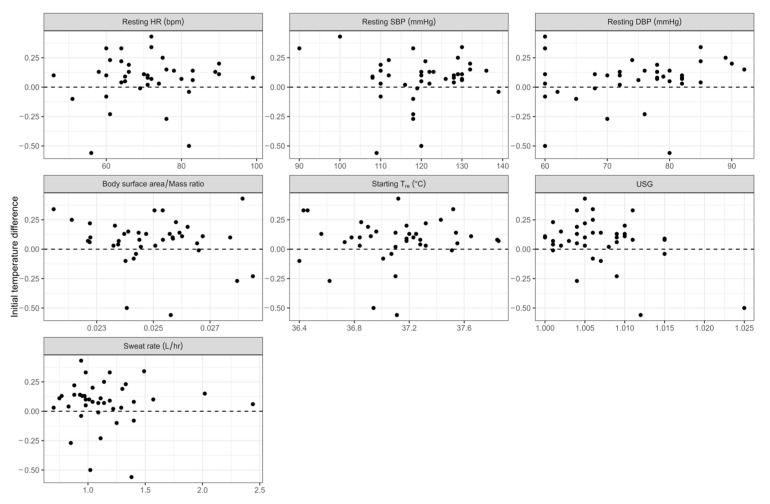
Relationship between the commencing temperature difference between gastrointestinal (T_gi_) and rectal (T_re_) core temperatures and each covariate. HR, heart rate; SBP, systolic blood pressure; DBP, diastolic blood pressure; USG, urine specific gravity.

To determine if any of the assessed variables influenced the difference in core temperature between the commencing T_re_ and T_gi_ temperatures several univariate linear regression analyses were conducted. The covariates assessed included resting HR, resting systolic and diastolic BP, BSA/mass ratio, commencing core temperature, baseline USG and sweat rate for the difference between T_re_ and T_gi_ at time zero of the HTT. Scatter plots showing the difference between T_re_ and T_gi_ and each variable are presented in Figure 6. None of the investigated parameters were significantly related to the difference in commencing T_re_ and T_gi_ (*p* > 0.05; Table 3).

### 3.4. Comparison of Covariates Between Heat-Tolerant and Heat-Intolerant Trials

To compare between heat-tolerant and heat-intolerant trials, the data was pooled according to heat tolerance outcome (as determined by T_re_ above 38.5 °C or HR above 150 bpm). The median (IQR) age, height and weight were 28.3 (21.5–32.2) and 25.1 (19.8–33.3) years, 177.5 (171.1–181.1) and 179.2 (174.6–181.0) cm, and 72.66 (70.02–79.56) and 85.40 (77.65–93.75) kg for heat-tolerant and heat-intolerant trials, respectively. Age and height did not differ significantly between the heat-tolerant and heat-intolerant trials (*p* > 0.05) but weight was significantly lower in the heat-tolerant compared to heat-intolerant trials (*p* = 0.002). Comparison between the heat-tolerant and heat-intolerant trials showed no significant differences between resting DBP, commencing core temperature (T_re_), baseline USG or sweat rate (Table 4). However, resting HR and SBP and final T_re_ were significantly lower and the BSA/mass ratio was significantly higher in the heat-tolerant trials compared to the heat-intolerant trials (Table 4).

## 4. Discussion

The HTT has been employed in a military context as a useful tool to assist with return to duty following EHS for many years. However, few studies have explored alternative measures to rectal temperature during the HTT. This study examined whether T_gi_ assessed via telemetric gastrointestinal core temperature pills could be used as an alternative to T_re_ during HTT. Although the two measures of core temperature showed good overall agreement during the HTT, there was only 80% agreement between T_gi_ and T_re_ for HTT outcome. There was also variable polarity in the bias between the two measures both between and within participant trials. We were unable to identify any covariates that could explain the differences between T_gi_ and T_re_. Furthermore, resting HR and systolic BP were significantly lower and the BSA/mass ratio was significantly higher in heat-tolerant trials compared to the intolerant trials.

Return to duty or play following EHS is a complex process [6,7]. In a military context, ensuring recovery from EHS is not only important for the individual, minimizing the risk of EHS reoccurrence, but also because each member of a military unit must be relied upon to be at peak capacity during combat. Heat tolerance testing is utilized by a number of Defence Forces around the world, including the Australian and Israeli Defence Forces, to indicate safety to return to duty following EHS. Although some controversy surrounds the use of the IDF HTT [28], it has been used by the IDF for over 30 years to assess readiness to return to duty following EHS [29] and is currently the only validated HTT protocol [30]. The IDF HTT has been suggested to be inappropriate for athletes [31] because of the walking protocol; however, it was appropriate in this study with a military focus. Indeed, the 5 km/h walking speed mimics a 10 km military march over 2 h.

Traditionally core temperature is assessed via rectal temperature during the HTT [9,10,11,32]. Therefore, T_gi_ was compared to T_re_ in this study despite previous research suggesting T_re_ is not an appropriate criterion measure for other core temperature measures due to the slow response time to changes in central blood temperature [15]. Our results showed that T_re_ was significantly lower than T_gi_ at time 0 of the HTT. However, there was good overall agreement between T_re_ and T_gi_ temperatures during the HTT with a median bias of 0.2 °C (95% LoA −0.66 to 0.39 °C) and Spearman’s rank correlation ρ = 0.893. These levels of agreement are consistent with past studies comparing these two core measures in a variety of contexts including outdoor team sport exercise in heat [18] (mean bias −0.19 °C; r = 0.86); during indoor exercise in hot conditions [20] (mean bias −0.02 °C; r = 0.86); during moderate intensity cycling in thermoneutral and hot conditions [21] (mean bias 0.02 °C; r = 0.92); during intermittent exercise [19] (mean bias −0.15 °C; r = 0.98) and during recovery from running in warm weather [22] (mean bias −0.06 °C; r = 0.78). Furthermore, the bias falls within the 0.27 °C levels suggested to be valid when allowing for inherent biological variation [18]. The significant difference in the two core measures observed at the start of the HTT in the current study was lost at test completion when heat tolerance status is diagnosed. The T_gi_ and T_re_ measures were in agreement on heat tolerance outcomes in 80% of the trials examined. In the remaining 20% of trials where T_gi_ and T_re_ were not in agreement, T_gi_ failed to detect heat intolerance identified by T_re_ in 6 of 8 trials. If T_gi_ were the only measure of core temperature during HTT this could result in returning military members to duty who are not necessarily heat-tolerant via the T_re_ measure, potentially placing them at risk of further EHS. There was no consistency in the polarity of the bias between the two core measures either within or between participant trials. This variable polarity makes it difficult to design an alternative protocol with a correction factor for a T_gi_ temperature threshold. We did investigate whether varying the threshold temperature of T_gi_ could bring the two core measures into a diagnostic agreement; however, this did not improve the agreement in HTT outcome. Therefore, on the basis of the 20% variability in heat tolerance outcomes between the two measures, we cannot recommend using T_gi_ in place of T_re_ during HTT.

We were unable to identify any of the measured covariates that could explain the significant difference in T_gi_ and T_re_ at HTT commencement. The data we had available included resting HR, resting systolic and diastolic BP, BSA/mass ratio, HTT commencing core temperature, baseline USG and sweat rate. None of these variables were related to the temperature differential between T_gi_ and T_re_. Rectal temperature is considered a reliable measure of core temperature during exercise testing [13,14]; however, as mentioned above, it has been reported to track temperature changes more slowly than other core temperature measures [15]. The HTT was developed over a number of years commencing in 1979 [33] when rectal temperature was one of the few reliable measures of human core temperature commonly available. Telemetric gastrointestinal core temperature pills have been available for human use for many years but have only been commonly used in exercise testing more recently providing an ability to assess core temperature during field testing. However, there are some limitations of the gastrointestinal pills. Sufficient time must be allowed for the core pill to transit from the stomach into the small intestine otherwise drinking may affect the measured temperature [34]. As the HTT is 2 h duration, it is not possible to prohibit drinking, and the ingestion of warm fluids could bias the test outcome. Therefore, sufficient time should be allowed between pill ingestion and exercise testing for the pill to transit out of the stomach. A period of 6–8 h was allowed in the current study consistent with other studies [35,36,37]. It should be noted that people with very high fibre diets may pass the pill in a 6–8 h time period, although this did not occur in the current study we have experienced this previously in our laboratory. Darwent et al. [38] reported the mean transit time through the body of 27.2 (±13.7; range 16.6–51.7) hours for 87 core temperature pills taken by 11 participants. During a 2 h HTT, the core pill will transit along the small intestine. It is therefore possible that the measured temperature from the core pill will be influenced by proximity to metabolically active organs such as the liver. It has been shown that a temperature gradient exists down the gastrointestinal tract [17] which will also influence the measured core temperature as the core pill moves through the bowel. A relatively longer time period between ingestion and testing may increase the likelihood of the gastrointestinal temperature more closely mapping the rectal temperature as the pill travels further through the bowel. It would be worthwhile investigating other factors such as the location of the core pill in the gastrointestinal track, transit time and dietary intake that may explain the temperature difference between T_re_ and T_gi_.

Other factors that should be considered in the use of the gastrointestinal core temperature pills include expense and reliability. The core pills are single-use only and not retrieved after use which increases the expense compared to rectal probes. The core pills are also susceptible to electrical interference and battery failure with previous studies noting incomplete or inaccurate data (see [17]). However, correct storage and treatment of the core pills should minimize pill failure and loss of data. Participants should therefore receive clear instructions on care of the pill prior to ingestion.

A heat-intolerant outcome occurred in 46% of the trials for the cases in this study. The time period between EHS and HTT was variable ranging from 6.9 to 68 weeks with one outlier referred almost 6 years after the EHS event. Thus, 54% of the cases in this study were sufficiently recovered to be classified as heat-tolerant on the HTT despite their previous EHS. Past research has shown that people experience a transient period of heat intolerance following EHS [39,40]. The HTT may be useful for identifying state versus trait heat intolerance [30]. People who have been given sufficient time to recover from EHS and then gradually improve their aerobic fitness (firstly in neutral environments followed by training in heat; see [41]) should obtain a heat-tolerant outcome on the HTT reflecting initial state heat intolerance. However, those people who still show heat intolerance despite adequate recovery and retraining may have trait heat intolerance and always be susceptible to heat illness. Controversy exists regarding state and trait heat intolerance [42] and this area requires further research, particularly into possible genetic factors influencing heat intolerance.

Resting HR and systolic BP were significantly lower and BSA/mass ratio significantly greater in the heat-tolerant trials compared to the intolerant trials in this study but commencing core temperature, resting diastolic BP, baseline hydration status (USG) and sweat rate were not significantly different between the tolerant and intolerant trials. Lower resting HR is associated with higher aerobic fitness as stroke volume increases with adaptations to endurance training [43,44]. Furthermore, higher aerobic fitness as assessed by VO_2max_ is associated with better heat tolerance [4,45,46,47]. Adaptations to heat are similar to those that occur during endurance training including increased plasma volume and earlier and greater sweating responses [48]. This likely results from the large amounts of metabolic heat produced by the contracting muscles during endurance exercise. Thus, regardless of the environment in which training takes place, heat tolerance is increased as a result of aerobic training [48]. Therefore, endurance training conveys heat tolerance which is supported by the lower resting HR observed in the heat-tolerant trials in this study. Furthermore, systolic BP is known to be reduced in individuals who undertake regular aerobic exercise training [49] which is consistent with the significantly lower systolic BP observed in the heat-tolerant trials in the current study, aligning with the lower resting HR finding. These findings have implications for military forces to ensure that Defence members meet adequate aerobic fitness entry requirements and maintain this aerobic capacity during military service as low aerobic fitness could place them at higher risk for heat illness.

Heat exchange with the environment is affected by the BSA/mass ratio [50]. Smaller, leaner people have a greater surface area for heat loss relative to mass compared to larger people with greater mass. Thus, children and small females are at greater risk of heat loss and hypothermia in cold environments [50]. In our study heat-tolerant participants had a significantly greater BSA/mass ratio compared to heat-intolerant participants, possibly due to their greater heat loss ability relative to mass. This finding supports previous research showing that people with lower BSA/mass ratio had an increased risk of EHS in healthy military populations [51], that the odds for heat intolerance on the IDF HTT was more likely to occur with every unit decrease in BSA/mass ratio [52] and a case study suggesting a low BSA/mass ratio as the most likely cause of heat intolerance [53]. The increasing rates of obesity around the world in the general population [54] have implications for military recruitment and training. Greater levels of obesity result in the lower BSA/mass ratio which could lead to a greater risk of heat illness. If recruiting standards are reduced in reference to percentage body fat this could put military members at higher risk of heat illness. Obesity was previously recorded as the top reason for ineligibility for military service in the USA Department of Defense [55]. Reduction in the physical standards for military recruitment could assist with increasing recruitment; however, this would need to be weighed up against an increased risk of heat injury.

Baseline hydration status as assessed by USG was not found to be significantly different between the heat-tolerant and -intolerant trials in this study. This is likely due to the low USG level required prior to commencing the HTT. Euhydration was required prior to the test with a USG level set at 1.015 or below. The starting core temperature was also not significantly different between the heat-tolerant and -intolerant trials. Therefore, there was no advantage to commencing the test with a lower core temperature in terms of heat tolerance outcome.

Sweat rate was also not significantly different between the heat-tolerant and -intolerant trials in this study. Sweat rates varied from 0.65 to 2.44 L/hr with the overall median (IQR) of 1.07 (0.94–1.26) L/hr. This is higher than sweat rates previously reported for the IDF HTT protocol with other studies (based in Israel) reporting sweat rates of 0.78 ± 0.16 L/hr [11] and 0.77 ± 0.28 for males and 0.52 ± 0.23 L/hr for females [52]. The higher sweat rate observed in the current study may relate to the recruitment of the majority of participants from tropical northern Australia, thus they were acclimatized to hot/humid conditions. Israel has a dryer climate compared to tropical northern Australia. Sweat suppression has been suggested to be reduced in people acclimatized to hot/humid conditions compared to hot/dry conditions [56], which may explain the higher sweat rates in our study. There was no significant difference in sweat rate between the heat-tolerant and heat-intolerant trials. Sweat rate increases with heat acclimatization [57,58] along with an earlier sweat onset [57]. However, in this study heat-tolerant participants had a significantly lower final core temperature at HTT completion (median (IQR) final T_re_ 37.99 (37.65–38.26) °C), as expected, compared to heat-intolerant participants (median (IQR) final T_re_ 38.59 (38.52–38.78) °C) which would thus decrease the need for heat-tolerant people to sweat during the HTT. The lower core temperature in heat-tolerant trials may thus explain the lack of significant difference in sweat rates between the heat-tolerant and -intolerant trials.

The control participants recruited to this study were healthy, physically active volunteers with no history of heat illness. However, 64% of the controls had heat-intolerant outcomes from the HTT which was not significantly different from the proportion of cases who were heat-intolerant (46%). The controls were recruited from the university population and local sporting groups. The four well-trained athletes who volunteered as controls showed very good heat tolerance (final HR below 121 bpm, final core temperature below 37.65 °C). All 14 controls took part in a concurrent study on heat tolerance which included assessment of VO_2max_ [24]. The four athletes had an average VO_2max_ of 57.2 ± 1.5 mL/kg/min whereas the controls recruited from the university had an average VO_2max_ of 39.6 ± 6.0 mL/kg/min with a greater focus on resistance training in this group rather than aerobic training. Notably, three of the controls could not complete the 2 h HTT protocol with core temperatures above the threshold and/or mild symptoms of heat illness including headache and dizziness when the test was ceased. The high proportion of heat-intolerant outcomes in the control group illustrates the importance of maintaining good aerobic fitness to decrease the risk of heat illness. This has further implications for the general population as physical inactivity levels have been reported to have increased by 5% from 2010 to 2022 [59]. If this decrease in physical activity is accompanied by decreases in aerobic fitness there will be an increased risk of heat illness during severe hot weather events. Furthermore, as global warming increases and the outside environment becomes less hospitable, people may be more likely to spend a greater proportion of their time in air conditioning, avoiding heat, and will thus decrease their heat acclimatization, putting them at further risk of heat illness.

This study was limited by the use of a civilian control group as access to ADF controls could not be accomplished because of the risk to military careers from a possible heat-intolerant outcome. However, comparison with the civilian controls provided further insight into the factors contributing to heat intolerance. Furthermore, we could not control for prior heat acclimatization as ADF participants were referred for testing from various locations in Australia. However, the majority (70%) were referred from hot/humid locations and all controls had experienced at least one summer in the testing location.

## 5. Conclusions

In conclusion, T_re_ and T_gi_ showed good agreement during HTT with Spearman’s rank correlation of 0.893 and median bias of 0.2 °C. However, the HTT outcome was only in agreement between the two core temperature measures in 80% of trials. In the remaining 20% of trials where T_re_ and T_gi_ did not agree, T_gi_ was more likely to indicate heat tolerance. This may result in an early return to duty following EHS and a higher risk of EHS reoccurrence if T_gi_ were the only measure of core temperature during HTT. We therefore do not recommend the use of T_gi_ in place of T_re_ during HTT. Our data also revealed that the BSA/mass ratio was significantly greater and resting HR and resting SBP were significantly lower in the heat-tolerant trials compared to the intolerant trials which may have reflected higher aerobic fitness for heat-tolerant participants. These findings have implications for military forces to maintain criteria for aerobic fitness and body composition to decrease the risk of serious heat illness.

## Figures and Tables

**Table 1:** Median (IQR) age, height, weight and BSA/mass ratio and proportion heat-intolerant of the cases and controls.

Characteristic	Cases (*n* = 26) *	Controls (*n* = 14)	*p*-Value
Age (years)	26.0 (21.3–33.0)	27.7 (18.9–34.4)	0.340
Height (cm)	178.0 (173.4–181.1)	178.5 (170.7–181.4)	0.888
Weight (kg)	78.0 (72.4–90.3)	81.5 (65.6–86.8)	0.461
BSA/mass ratio	0.0250 (0.0237–0.0257)	0.0248 (0.0242–0.0272)	0.307
Heat-intolerant (*n*) ^#^	12 (46.2%)	9 (64.3%)	0.333

* *n* = 26 for all variables except height where *n* = 23 as three cases undertook two trials and height did not vary between trials; # heat tolerance status determined from criteria Tre > 38.5 °C or HR > 150 bpm [12]; IQR, interquartile range; BSA, body surface area.

**Table 2:** Confusion matrix showing heat tolerance test outcome compared by T_re_ and T_gi_ criteria.

	Heat Tolerance Outcome by T_gi_ (Failed HTT by T_gi_)		
**Heat tolerance outcome by T_re_** **(Failed HTT by T_re_)**		False (Tolerant)	True (Intolerant)
	False (Tolerant)	20	2
	True (Intolerant)	6	12

HTT, heat tolerance test; T_gi_, gastrointestinal core temperature; T_re_, rectal core temperature.

**Table 3:** Univariate linear regression analysis for covariates on the difference between commencing T_gi_ and T_re_.

Covariate	Regression Coefficient	95% Confidence Intervals	*p*-Value
Resting HR (bpm)	0.00121	−0.00462 to 0.00704	0.676
Resting SBP (mmHg)	−0.000399	−0.00683 to 0.00604	0.901
Resting DBP (mmHg)	0.00289	−0.00406 to 0.00984	0.405
BSA/mass ratio	−19.1	−58.2 to 20.0	0.329
Commencing core temperature (T_re_; °C)	0.0356	−0.147 to 0.218	0.695
Baseline USG ^#^	−7.337	−21.4 to 6.69	0.296
Sweat rate (L/hr)	−0.00583	−0.204 to 0.193	0.953

HR, heart rate; SBP, systolic blood pressure; DBP, diastolic blood pressure; BSA, body surface area; T_re_, rectal core temperature; USG, urine specific gravity; *n* = 39 trials due to one core pill malfunction on an initial data point; ^#^ *n* = 39 due to one missing value.

**Table 4:** Median (IQR) resting HR, resting SBP and DBP, BSA/mass ratio, commencing and final core temperature, baseline USG and sweat rate for heat-tolerant and intolerant trials.

Characteristic	Heat-Tolerant (*n* = 19)	Heat-Intolerant (*n* = 21)	*p*-Value
Resting HR (bpm)	66.0 (60.5–73.0)	75.0 (71.0–83.0)	0.0154
Resting SBP (mmHg)	118.0 (110.0–121.0)	128.0 (118.0–130.0)	0.0465
Resting DBP (mmHg)	76.0 (68.0–79.5)	78.0 (70.0–82.0)	0.473
BSA/mass ratio	0.0257 (0.0249–0.0266)	0.0241 (0.0228–0.0250)	0.00133
Commencing core temperature (T_re_; °C)	37.10 (36.85–37.18)	37.25 (36.96–37.52)	0.0830
Final core temperature (T_re_; °C)	37.99 (37.65–38.26)	38.59 (38.52–38.78)	<0.001
Baseline USG ^#^	1.007 (1.004–1.010)	1.006 (1.003–1.008)	0.384
Sweat rate (L/hr)	1.04 (0.96–1.22)	1.09 (0.88–1.40)	0.626

HR, heart rate; SBP, systolic blood pressure; DBP, diastolic blood pressure; BSA, body surface area; T_re_, rectal core temperature; USG, urine specific gravity; ^#^ *n* = 39 due to one missing heat-intolerant value.

## Data Availability

The data analyzed and presented in this study are included in this published article. The raw data are not available as part of the data was obtained from military personnel and the parameters of the ethics approval do not allow data sharing. Further enquiries can be directed to the corresponding author.

## Abbreviations

The following abbreviations are used in this manuscript:

ADF	Australian Defence Force
BP	Blood pressure
BSA	Body surface area
DBP	Diastolic blood pressure
EHS	Exertional heat stroke
HR	Heart rate
HTT	Heat tolerance test
IDF	Israeli Defence Force
IQR	Interquartile range
LoA	Limits of agreement
SBP	Systolic blood pressure
SR	Sweat rate
T_gi_	Gastrointestinal core temperature
T_re_	Rectal core temperature
USG	Urine specific gravity
VO_2max_	Maximal oxygen consumption
